# Serum amyloid A in cats with renal azotemia

**DOI:** 10.14202/vetworld.2023.1673-1681

**Published:** 2023-08-19

**Authors:** Laura Degenhardt, Roswitha Dorsch, Katrin Hartmann, René Dörfelt

**Affiliations:** LMU Small Animal Clinic, Center for Clinical Veterinary Medicine, LMU München, Veterinärstraße 13, 80539, Munich, Germany

**Keywords:** acute kidney injury, acute-phase protein, chronic kidney disease, International Renal Interest Society grading, uremia

## Abstract

**Background and Aim:**

The concentration of the feline acute-phase protein serum amyloid A (SAA) increases in cats with acute inflammatory diseases. However, it is unclear whether SAA concentration increases in cats with azotemic kidney disease or whether it can aid in differentiating acute kidney injury (AKI) from chronic kidney disease (CKD). Similarly, whether SAA concentration can be used as a prognostic marker is also unclear. Therefore, this study aimed to evaluate the SAA concentrations in cats with azotemic kidney disease and determine whether SAA concentrations can be used to differentiate between AKI, CKD, and “acute on CKD” (AoC). In addition, we evaluated whether SAA concentration could serve as a prognostic parameter. Moreover, we determined the correlations between SAA concentration and temperature; creatinine, urea, and albumin concentrations; leukocyte count; and urine protein/creatinine (UP/C).

**Materials and Methods:**

Forty-eight client-owned azotemic cats (creatinine >250 μmol/L) were included in this prospective study. Cats with pre- and post-renal azotemia were excluded from the study. The causes of azotemia were differentiated into AKI, CKD, and AoC. The SAA concentrations were analyzed through turbidimetric immunoassay at the time of admission. Data were analyzed using the Mann–Whitney U, Kruskal–Wallis, Chi-Square, Fisher’s exact, and Spearman correlation tests. p ≤ 0.05 was considered statistically significant.

**Results:**

Serum amyloid A concentration increased in 5/12 cats with AKI, 7/22 cats with CKD, and 9/14 cats with AoC (p = 0.234). The median SAA concentration in cats with AKI, CKD, and AoC whose SAA concentration was ≥5 mg/L was 174 mg/L (10–281 mg/L), 125 mg/L (6–269 mg/L), and 143 mg/L (7–316 mg/L), respectively (p = 0.697), with no significant differences observed between the groups. The median SAA concentration did not differ significantly between survivors (125 mg/L, 10–316 mg/L) and non-survivors (149 mg/L, 6–281 mg/L; p = 0.915) with SAA concentration ≥5 mg/L.

**Conclusion:**

Serum amyloid A concentration increased in 44% of the cats with azotemia. However, it cannot be used to differentiate AKI from CKD or as a prognostic marker. Serum amyloid A concentration was correlated with neutrophil count, albumin concentration, and UP/C, and the presence of comorbidities may influence SAA concentration.

## Introduction

Feline serum amyloid A (SAA), a major acute-phase protein (APP), is an apolipoprotein complexed with high-density lipoproteins. Serum amyloid A is produced in the liver in response to pathogenic stimuli [1, 2]. Serum amyloid A synthesis is modulated by macrophages through the release of cytokines, such as interleukin (IL)-1, IL-6, and tumor necrosis factor-alpha (TNF-α). Glucocorticoids can also enhance the synthesis of SAA [2, 3]. In cats, SAA reaches its peak concentration, up to 10–50 times the reference range, 24–48 h after exposure to a pathogenic stimulus. In addition, the concentration remains high throughout the duration of inflammation [1, 2, 4]. Serum amyloid A has an immunomodulatory activity, and it can decrease inflammatory cell damage and oxidize cholesterol by acting as an oxygen radical scavenger [2, 5].

Serum amyloid A is a known prognostic marker for many feline diseases where the increase in its concentration is a decisive factor [6]. In addition, it can also be used for monitoring the response to treatment in cats with pancreatitis and feline infectious peritonitis [7, 8].

An increase in the concentration of SAA has also been observed in non-inflammatory diseases, such as renal diseases, neoplasia, and diabetes mellitus [9–12]. Chronic kidney disease (CKD) is defined as a structural and/or functional impairment of one or both kidneys for ≥3 months [13]. A previous study by Kongtasai *et al*. [14] reported that CKD prevalence in cats ranges from 2% to 4%, increasing to 40% in cats over 10 years. Serum amyloid A concentration increases mildly in cats with CKD. However, no significant differences are observed between the SAA concentrations of patients with the International Renal Interest Society (IRIS) CKD stages 2–4 [15].

An abrupt decline in renal function leading to a decrease in the glomerular filtration rate and urine production is defined as acute kidney injury (AKI) [16]. AKI can be caused by insults, such as infections, drugs, toxins, and ischemia [16]. Legatti *et al*. [17] reported a mortality rate of 53.1% in cats with AKI in their meta-analysis. An acute decrease in renal function in formerly stable CKD is defined as “acute on CKD” (AoC), and its long-term survival of AoC is guarded, with a median survival time of 66 days [18].

Chronic kidney disease and AKI can be differentiated based on the history, physical examination findings, laboratory blood and urine values, and ultrasonographic appearance of the kidneys. Therefore, the IRIS has developed guidelines to assist in the diagnosis, staging, and treatment of CKD and AKI. Chen *et al*. [18] reported in their study that the clinical presentation of AoC resembled that of AKI; thus, differentiating between AoC and AKI is difficult. Thus, as there are no reliable parameters that can assist in differentiating between AKI, CKD, and AoC, differentiating between AKI, CKD, and AoC remains challenging.

This study aimed to investigate SAA concentrations in cats with azotemic kidney disease with CKD IRIS stages 3–4 and AKI IRIS grades 3–5 and to determine whether SAA concentration can be used to differentiate between AKI, CKD, and AoC. Furthermore, we also evaluated whether SAA concentration can be used as a prognostic parameter. In addition, we determined the correlations of SAA with temperature; creatinine, urea, and albumin concentrations; leukocyte count; and urine protein/creatinine (UP/C).

## Materials and Methods

### Ethical approval

The study protocol was approved by the Ethics Committee of the Center for Clinical Veterinary Medicine (number: 68–19–05–2016), LMU Munich.

### Study period and location

This study was conducted from July 2017 to January 2019 at the Center for Clinical Veterinary Medicine, LMU Munich.

### Study design

The prospective study included 48 client-owned cats who presented to the Emergency Department of the Center for Clinical Veterinary Medicine with serum creatinine concentrations >250 μmol/L. Cats with pre- and post-renal azotemia were excluded from the study. Cats who had received prior treatment and those with comorbidities were not excluded from this study because cats are often brought to the study center for a second opinion. The history was obtained at the presentation, and a full physical examination was performed. All cats were hospitalized for further diagnostic examinations and treatment.

Abdominal ultrasonography (Logiq P6, GE Healthcare, Chalfont St. Giles, United Kingdom) was performed by a Diplomate ECVIM-CA. The complete blood count, including platelet counts (Sysmex XT-2000i; Sysmex Corporation, Kobe, Japan), serum chemistry (COBAS INTEGRA 400 plus; F. Hoffmann-La Roche AG, Basel, Switzerland), and blood gas analysis (RAPIDPoint 450; Siemens AG, Berlin, Germany), was performed at presentation. In addition, urinalysis, including urine specific gravity, urine stick analysis (Combur 5 Sticks; F. Hoffmann-La Roche AG, Basel, Switzerland), urine sediment, and UP/C ratio (COBAS INTEGRA 400 plus; F. Hoffmann-La Roche AG) was performed.

**Figure-1:**
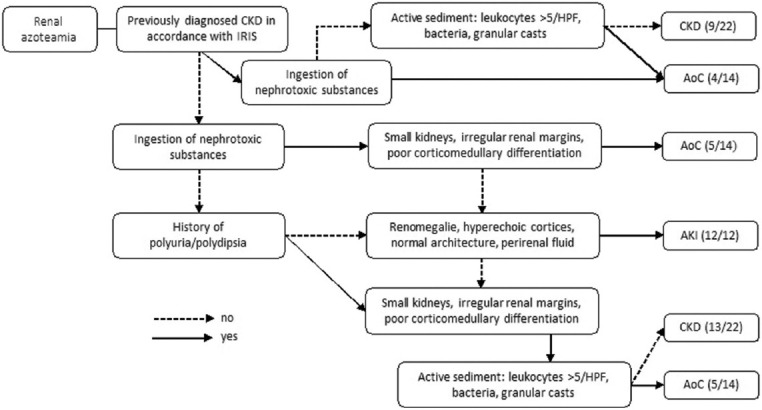
Flow chart for the classification of AKI, CKD and AoC in cats. SAA=Serum amyloid A, AKI=Acute kidney injury, CKD=Chronic kidney disease, AoC=Acute on chronic kidney disease, HPF=High-power field, IRIS=International Renal Interest Society.

Cats with pre- or post-renal azotemia were excluded based on the history and results of urinalysis and ultrasonography. Cats with a urine specific gravity of >1.035 were categorized as having pre-renal azotemia. In contrast, those with urethral obstruction, ureteral obstruction with pyelectasia of >3.5 mm, or ureteral distension of >1 mm observed during sonography were categorized as having post-renal azotemia [19, 20]. International Renal Interest Society staging/grading was performed using the results of history, physical examination, serum creatinine concentrations, other laboratory values, urinalysis, and sonography. The cats were subsequently classified into the AKI, CKD, or AoC groups (Figure-1). Patient survival until discharge was recorded, and patients who died or were euthanized during the study period were classified as non-survivors.

### Samples

Blood samples were collected at the presentation for further analysis, and the samples were frozen at −18°C and sent for SAA analysis to a commercial laboratory within the same week. Serum amyloid A concentrations were measured using a turbidimetric immunoassay (LZ-SAA Standard Q; product code: G-SZ75; Eiken Chemical Co., LTD., Tokyo, Japan) with an assay linearity of 5–500 mg/L and a reference value <5 mg/L, analyzed with a chemistry analyzer (AU 5800; Beckman Coulter Inc., Brea, USA). The test kit was based on the principle of a latex agglutination reaction. The latex particles were sensitized with anti-human SAA rabbit polyclonal and anti-human SAA mouse antibodies, with the change in turbidity corresponding to the SAA concentration. The LZ-SAA test kit was validated by Hansen *et al*. [21] and verified by Tamamoto *et al*. [22].

### Statistical analysis

Statistical analyses were performed using commercial software (Prism Windows 5; GraphPad Software, San Diego, California). Normality was analyzed using the D’Agostino and Pearson normality tests. Parametric and normally distributed data were presented as mean ± standard deviation, whereas non-parametric and non-normally distributed data were presented as medians and ranges.

The Mann–Whitney U and Chi-square tests were used to analyze the differences between the SAA concentrations of survivors and non-survivors as well as for the intragroup comparison of the SAA concentration in the AKI, CKD, and AoC groups with respect to comorbidities. The differences between the SAA concentrations in the AKI, CKD, and AoC groups were analyzed using the Kruskal–Wallis and Chi-square tests. Fisher’s exact test was used to analyze the association between gender and SAA. Moreover, the correlations between SAA and temperature; creatinine, urea, and albumin concentrations; leukocyte, neutrophil, and band neutrophil counts; and UP/C were analyzed using the Spearman correlation test. p ≤ 0.05 was considered statistically significant.

## Results

### Study population

The mean age and mean body weight of the 48 cats included in the study were 11.9 ± 5.5 years and 3.9 ± 1.4 kg, respectively. Gender distribution was as follows: 20/48 neutered males, 18/48 spayed females, 5/48 intact males, and 5/48 intact females. Most cats were domestic shorthair cats (40/48). The other breeds included Persian (3/48), Ragdoll, Bengal, Birman, Siamese, and British shorthairs (1 cat each).

Twelve cats were diagnosed with AKI, 22 cats were diagnosed with CKD, and 14 cats were diagnosed with AoC (Table-1). The cause of AKI and AoC was identified in 18 cats (intoxication/drugs 10; neoplasia 4; and pyelonephritis 4), whereas it remained unknown in eight cats (Table-2). The presence of comorbidities was also documented for all cats (Table-3).

**Table-1:** Number of cats with azotemic kidney disease with normal versus increased SAA concentration.

	All	AKI	CKD	AoC	Survivors	Non-survivors
Total	48	12	22	14	27	21
SAA <5 mg/L	27	7	15	5	18	9
SAA ≥5 mg/L	21	5	7	9	9	12

There was no significant difference between the SAA concentration of the AKI, CKD, and AoC groups (p = 0.234). There was no significant difference between the SAA concentration of the survivors and non-survivors (p = 0.144). SAA=Serum amyloid A, AKI=Acute kidney injury, CKD=Chronic kidney disease, AoC=Acute on chronic kidney disease

**Table-2:** IRIS grade and cause of azotemia in 18 cats with AKI and AoC.

Total	IRIS grade (2016)			Cause of azotemia				
	
	3	4	5	Neo	Intox	Pyelonephritis	Drugs	Unknown
AKI								
12	2	6	4	1	1	-	5	5
AoC								
14	6	4	4	3	1	4	3	3

IRIS=International Renal Interest Society, AKI=Acute kidney injury, AoC=Acute on chronic kidney disease, Neo=Neoplasia, Intox=Intoxication

**Table-3:** Signalment, comorbidities, survival, and selected clinicopathological data of 48 azotemic cats.

Number	Age (years)	Gender	SAA (mg/L)	Creatinine (μmol/L)	WBC counts (G/L)	Neutrophil count (G/L)	Cause of azotemia	AKI/CKD/AoC	Comorbidity	Survival
1	12	F	315.5	972	9.2	6.6	Drugs	AoC	None	Yes
2	0	F	281.4	762	10.9	8.2	Drugs	AKI	None	No
3	14	M	268.6	511	17.7	15.2	Unknown	CKD	HHS	No
4	0	F	246.4	539	20.4	18.9	Drugs	AKI	Polyneuropathy	Yes
5	12	F	242.5	1395	25.3	24.8	Neoplasia	AoC	Pyometra	No
6	13	F	212.7	274	5.8	2.8	Unknown	CKD	Perforated intestinal foreign body, pancreatitis	No
7	18	F	180.9	501	28.2	24.9	Pyelonephritis	AoC	Pancreatitis	Yes
8	12	F	173.6	484	15.7	15.1	Unknown	AKI	Dermal lesions	No
9	17	F	160.0	367	21.4	18.3	Pyelonephritis	AoC	Hyperthyroidism	Yes
10	11	M	153.3	255	5.2	3.4	Unknown	CKD	DKA	No
11	18	F	143.3	852	16.8	15.7	Unknown	AoC	None	No
12	4	M	124.6	310	17.5	Nd	Unknown	CKD	Diarrhea	Yes
13	11	M	124.6	2460	21.7	20.9	Drugs	AKI	None	No
14	19	F	119.9	283	18.1	15.2	Pyelonephritis	AoC	Neoplasia urinary bladder	Yes
15	19	F	118.0	282	26.8	22.2	Pyelonephritis	Aoc	Pancreatitis	Yes
16	19	F	26.5	286	7.4	5.2	Unknown	CKD	Hyperthyroidism	Yes
17	9	M	14.6	978	16.3	14.7	Intoxication	AoC	None	No
18	10	M	13.3	352	10.5	10.1	Unknown	CKD	DKA	No
19	8	M	9.7	733	9.7	8.1	Unknown	AKI	Enteropathy	Yes
20	14	F	6.6	977	11.7	10.6	Unknown	AoC	Pancreatitis	No
21	12	F	5.9	342	21.9	15.2	Unknown	CKD	Epilepsy	No
22	16	M	<5.0	530	13.5	9.4	Neoplasia	AoC	Cystitis	Yes
23	8	M	<5.0	688	10.3	8.5	Neoplasia	CKD	None	Yes
24	-	F	<5.0	293	10.8	7.5	Unknown	CKD	None	Yes
25	2	M	<5.0	698	6.1	3.2	Neoplasia	CKD	None	Yes
26	19	M	<5.0	329	9.6	8.3	Unknown	CKD	Enteropathy	Yes
27	11	M	<5.0	667	7.2	6.3	Unknown	AKI	None	No
28	11	M	<5.0	1282	19.8	17.7	Unknown	CKD	Enteropathy	No
29	15	M	<5.0	394	11.0	9.6	Unknown	AoC	Peritonitis	No
30	16	M	<5.0	359	12.8	12.3	Unknown	CKD	Hyperthyroidism	No
31	4	M	<5.0	1771	11.4	7.7	Intoxication	AKI	None	No
32	12	M	<5.0	251	13.2	10.4	Neoplasia	CKD	None	Yes
33	15	F	<5.0	490	11.5	10.5	Hypoperfusion	CKD	Cardiomyopathy	No
34	9	M	<5.0	253	10.0	9.0	Drugs	AKI	Pancreatitis	Yes
35	9	F	<5.0	285	10.7	7.2	Unknown	CKD	Hepatopathy	No
36	3	M	<5.0	10221	29.8	14.2	Neoplasia	AKI	None	Yes
37	16	F	<5.0	451	16.5	14.4	Neoplasia	AoC	Pancreatitis	Yes
38	13	M	<5.0	317	16.9	12.8	Pyelonephritis	CKD	Cystitis	Yes
39	16	F	<5.0	334	14.8	11.6	Unknown	CKD	DKA	Yes
40	11	M	<5.0	345	9.0	6.2	Drugs	AoC	None	Yes
41	17	M	<5.0	358	8.7	5.5	Unknown	CKD	Cardiomyopathy	Yes
42	10	M	<5.0	958	14.5	13.0	Unknown	AKI	Cardiomyopathy	No
43	7	F	<5.0	778	20.6	17.9	Unknown	AKI	None	No
44	14	M	<5.0	251	9.4	7.1	Unknown	CKD	None	Yes
45	20	F	<5.0	251	10.3	6.6	Unknown	CKD	None	Yes
46	21	M	<5.0	273	11.5	9.8	Unknown	CKD	None	Yes
47	1	F	<5.0	261	17.2	13.4	Drugs	AKI	Cardiomyopathy	Yes
48	11	F	<5.0	353	9.5	6.3	Drugs	AoC	Peripheral vestibular syndrome	Yes

SAA=Serum amyloid A, WBC=White blood cell count, AKI=Acute kidney injury, CKD=CHRONIC kidney disease, AoC=Acute on chronic kidney disease, F=Female, M=Male, HHS=Hyperosmolar hyperglycemic state, DKA=Diabetic ketoacidosis, nd=No differential available

### Serum amyloid A concentration in cats with azotemic kidney disease

All cats had a median SAA concentration of <5 mg/L (<5–316 mg/L). An increase in the SAA concentration was observed (≥5 mg/L) in 21 cats, whereas it remained below the detection limit (<5 mg/L) in 27 cats. The median detectable SAA (≥5 mg/L) concentration for all 21 cats was 143 mg/L (6–316 mg/L).

**Figure-2:**
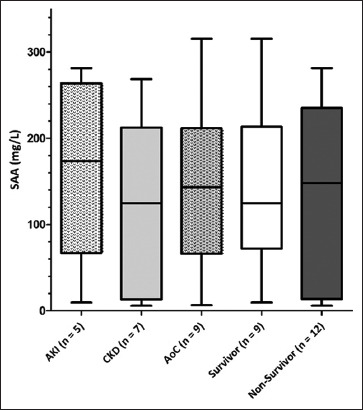
Median detectable SAA concentrations in 21 cats with renal azotemia. SAA=Serum amyloid A, AKI=Acute kidney injury, CKD=Chronic kidney disease, AoC=Acute on chronic kidney disease.

The median SAA concentrations were <5 mg/L (<5–281 mg/L), <5 mg/L (<5–269 mg/L), and 66 mg/L (<5–316 mg/L) in cats with AKI, CKD, and AoC, respectively (p = 0.167). The concentrations increased in 5/12, 7/22, and 9/14 cats with AKI, CKD, and AoC, respectively (p = 0.234; Table-1). The median SAA concentration in cats with AKI, CKD, and AoC whose SAA was ≥5 mg/L was 174 mg/L (10–281 mg/L), 125 mg/L (6–269 mg/L), and 143 mg/L (7–316 mg/L), respectively (p = 0.697; Figure-2).

### Intragroup comparison of SAA concentration for comorbidities

There was no significant difference between the median SAA concentration of cats with and without comorbidities in the AKI (p = 1.000) and AoC (p = 0.613) groups. However, a significant difference in the median SAA concentration was observed in the CKD group (p = 0.033). The median SAA concentration of cats with and without comorbidities in group CKD was <5 mg/L (<5–269 mg/L) and <5 mg/L (<5 mg/L–<5 mg/L), respectively.

### Serum amyloid A as a prognostic parameter

The median SAA concentration in the 27 surviving cats was 7 mg/L (<5–281 mg/L), whereas that in the 21 non-survivors was <5 mg/L (<5–316 mg/L). The SAA concentration was increased in 9/27 surviving and 12/21 non-surviving cats; however, 18/27 surviving and 9/21 non-surviving cats had SAA concentrations <5 mg/L (p = 0.144; Table-1). The median SAA concentration of survivors (125 mg/L, 10–316 mg/L) and non-survivors (149 mg/L, 6–281 mg/L; p = 0.915; Figure-2) whose SAA was ≥5 mg/L did not differ significantly.

Increased SAA concentrations were observed in 5/10, 1/7, and 4/5 cats with intoxication/drug use, renal neoplasia, and pyelonephritis, respectively (p = 0.437). In addition, female cats were significantly more likely to have increased SAA concentrations (14/23) than male cats (7/25; p = 0.041).

No correlation was observed between SAA concentration and temperature, creatinine and urea levels, and white blood cell and band neutrophil counts. However, a significant correlation was observed between SAA concentration and neutrophil count (r = 0.314; p = 0.032), albumin concentration (r = −0.354; p = 0.015), and UP/C (r = 0.548; p <0.001; Table-4).

**Table-4:** Correlation between SAA concentration and body temperature; albumin, creatinine, and urea concentrations; leukocyte, neutrophil, and band neutrophil counts; and UP/C in 48 cats analyzed using Spearman correlation test.

Parameter	r	p
Temperature	−0.168	0.265
Alb	−0.354	0.015
Creatinine	0.188	0.200
Urea	0.179	0.224
Leukocyte counts	0.240	0.101
Neutrophil count	0.314	0.032
Band neutrophil counts	0.512	0.061
UP/C	0.548	<0.001

SAA=Serum amyloid A, UP/C=Urine protein/creatinin ratio, Alb=Albumin

## Discussion

This study aimed to investigate the SAA concentration in cats with azotemic kidney disease with CKD IRIS stages 3 and 4 and AKI IRIS grades 3–5. In addition, this study also evaluated whether SAA concentration can be used for differentiating between AKI, CKD, and AoC.

Serum amyloid A concentration increased in 21/48 cats (44%), including 42% of the cats with AKI, 32% with CKD, and 64% with AoC, with no significant differences among the three groups. It is well known that chronic inflammation is associated with elevated concentrations of proinflammatory cytokines, such as IL-1, IL-6, and TNF-α, in humans with end-stage renal failure [23, 24]. Similarly, the progressive interstitial inflammation in CKD, inflammatory reaction triggered by renal tubular cell injury, ischemia in AKI, and reduced renal excretion of proinflammatory cytokines due to impaired renal function may increase the concentration of circulating acute APPs, such as SAA, in cats [25–27]. However, an increase in SAA concentration was not a consistent finding in this study. Among the 48 cats, 23 had received antimicrobial or anti-inflammatory therapy, such as steroids or non-steroidal anti-inflammatory drugs (NSAIDs), before presentation, which could have reduced the inflammation and SAA concentration. The Center for Clinical Veterinary Medicine acts as a referral center. Consequently, many cats presenting to the center for a second opinion had already received treatment from the primary veterinarian. Furthermore, NSAIDs can potentially induce AKI; therefore, their effect could not be excluded. Antibiotic treatment would influence the SAA concentration only if the elevation was caused by a bacterial infection. However, several antibiotics, such as penicillins, cephalosporins, and aminoglycosides, are known to cause AKI [16]. Thus, their nephrotoxic side effect could not be ruled out. Only three cats received corticosteroids in this study. However, the SAA concentration was >100 mg/L in only two of these cats, indicating that the influence of corticosteroids on the SAA concentration was negligible.

The duration of inflammation before presentation may also have played a crucial role in detecting SAA concentration using a single measurement. For instance, Kajikawa *et al*. [1] induced inflammation in cats through the intramuscular injection of turpentine oil and lipopolysaccharides (LPS) from *Escherichia coli* and reported that the SAA concentration peaked 24–48 h after the injection and decreased over the next 36–120 h. Thus, it can be hypothesized that presentation later in the disease and anti-inflammatory therapy could have resulted in a lower SAA concentration.

The cause of AKI/AoC was identified in 18 cats; ten had elevated SAA concentrations among these cats. One cat suffered from lily intoxication, three had received meloxicam, and one had received ibuprofen pre-azotemia. Lilies and NSAIDs are known to cause AKI [28–30]. Non-steroidal anti-inflammatory drugs inhibit cyclooxygenase, thereby reducing renal blood flow by interfering with the production of prostaglandins, resulting in ischemia, hypoxemia, and renal tubular cell injury [31]. Differences in dosage and/or other predisposing factors, such as hypoperfusion due to shock or anesthesia, could explain the increase in SAA concentrations in only 4/8 cats with drug-induced AKI/AoC.

Comorbidities were in 31/48 cats. Over the study period of two years, 70/89 cats with AoC and 34% of cats with CKD had comorbidities [15, 18]. Thus, the inclusion of cats without any comorbidities in an adequate number over a reasonable period was not feasible.

Sixteen of the 21 cats with increased SAA concentrations had comorbidities. Fifteen of these 21 cats had SAA concentrations >100 mg/L (118–316 mg/L). Among them, 11 cats had comorbidities, such as neoplasia, pyometra, diabetic ketoacidosis (DKA), hyperthyroidism, perforated intestinal foreign body, hyperosmolar syndrome, and polyneuropathy (Table-3), which were associated with higher SAA concentrations in previous studies. In humans, hyperosmolar non-ketotic coma and DKA are associated with an increased risk of sepsis, systemic inflammatory response syndrome, and elevation of APP C-reactive protein [32]. A case report on the consecutive monitoring of SAA in a cat with pancreatitis reported similar findings, with the cat displaying acute onset of symptoms with a concurrent increase in SAA concentration (28.3–153.5 mg/L) occurring four times [7]. Pancreatitis is a common comorbidity and cause of AKI in dogs and has been observed in 54% of cats with AoC [18, 33, 34]. Thus, the influence of pancreatitis on the development of AKI and AoC cannot be ruled out. In queens with pyometra and coexisting endometrial adenocarcinoma, the median SAA concentration was 32.3 μg/mL (0.4–83.9 μg/mL) [35]. Similarly, Tamamoto *et al*. [6] reported increased SAA concentrations in cats with neoplasia and hyperthyroidism.

Comorbidities did not significantly influence the SAA concentration in the AKI and AoC groups in the present study; however, a significant influence was observed in the CKD group. Moreover, SAA concentration did not differ between healthy cats and cats with CKD after excluding comorbidities [15]. Notably, although comorbidities may have contributed to the SAA concentration, particularly in CKD, in the present study, 5/21 cats had increased SAA concentrations without comorbidities (Table-3).

In this study, the median SAA concentration was below the detection limit (<5 mg/L) and lower than that reported in previous studies. In a previous study by Javard *et al*. [15], the mean SAA concentration in cats with CKD, including IRIS stages 2–4, was 8.1 ± 6.5 μg/mL, whereas the mean SAA concentration in healthy cats was 4.3 ± 1.2 μg/mL. In an azotemic subgroup of cats with various diseases, the mean SAA concentration was 31 ± 56 μg/mL [9]. Sandwich enzyme-linked immunosorbent assay (ELISA) and direct ELISA were used in the first and second studies, respectively [9, 15]. Kajikawa *et al*. [1] obtained an exceedingly high mean SAA concentration (118 ± 4 μg/mL) using sandwich ELISA in cats with induced inflammation through intramuscular injection of LPS. However, the median SAA concentration was not reported in this study. Other studies that used turbidimetric immunoassay reported an SAA concentration of 153.5 mg/L in a cat with pancreatitis and a median concentration of 18.1 mg/L in cats with neoplasia, inflammation, and other diseases, with a reference range of <0.9 mg/L and ≤0.82 mg/L, respectively [6, 7]. Variations in the SAA concentration are observed even in healthy control cats, with values ranging from 0.6–74.4 μg/mL [1, 9, 15, 36]. Thus, comparing the measured results is difficult due to the possible deviations caused by different test methods, as reported by Sasaki *et al*. [9], non-standardized reference limits of feline SAA, and variations in SAA concentrations in healthy cats.

The SAA concentrations of survivors and non-survivors with SAA ≥5 mg/L were similar; thus, SAA was not a useful prognostic marker in the present study population. A previous study by Yuki *et al*. [10] that included 444 cats with various diseases reported that SAA concentrations did not correlate with 30-day survival. Moreover, no correlation between SAA and prognosis could be identified in cats with lymphoma [37]. In contrast to these findings, in a previous study by Tamamoto *et al*. [6], 65 cats with various diseases and an elevated SAA concentration (>0.82 mg/L) had a shorter median survival time (72 days) compared with 110 cats with various diseases and a low SAA concentration (571 days).

This study observed a significant correlation between SAA concentration and neutrophil counts (Table-4). This finding may be attributed to cytokines that induce SAA synthesis and neutrophil release into the circulation. In addition, IL-1 and cortisol activate the marginal pool of neutrophils and induce a quick increase, whereas IL-1 and TNF-α simultaneously activate the bone marrow production of neutrophils [4]. A previous study by Tamamoto *et al*. [22] that evaluated SAA concentration in cats with multiple diseases reported a weak correlation between SAA, white blood cell count (r = 0.25), and band neutrophils (r = 0.08).

A significant negative correlation was observed between SAA and albumin levels, which can be attributed to two factors. First, hepatic synthesis of the negative APP albumin was possibly reduced to provide amino acids for the synthesis of positive APPs, for example, SAA [4]. Second, increased renal albumin loss may have also led to a negative correlation. Moreover, albumin is the main protein in the urine of healthy and sick cats. The significant correlation between SAA and UP/C indicated that UP/C is more sensitive to the detection of albumin than other proteins [38].

Kann *et al*. [39] reported a negative correlation between SAA and albumin (r = −0.444), with no significant difference observed between the median albumin concentrations of healthy (27.2 g/L) and sick cats (29.0 g/L).

In this study, female cats were significantly more likely to have increased SAA concentrations than male cats. Previous reports on gender-related differences in SAA concentrations are controversial. For instance, Kajikawa *et al*. [1] and Yuki *et al*. [10] reported no significant difference between the SAA concentrations of either gender. In contrast, Kann *et al*. [39] reported higher SAA concentrations in female cats.

In this study, the samples were stored at −18°C before undergoing a weekly batch analysis in the laboratory. Although stability studies of feline SAA have not been published, previous studies by Hillström *et al*. [40] and McDonald *et al*. [41] have reported that equine and human SAA concentrations remained stable at 4°C for 17 and 30 days, respectively. In contrast, bovine SAA concentration decreased on day 14 when stored at −18°C [42]. Therefore, storage at −18°C should not have impacted the SAA concentration in this study.

Nevertheless, this study had some limitations. A major limitation was that SAA concentrations below the assay range of the applied method were determined as <5 mg/L. Thus, determining numerical values below the assay range often produced negative results that could not be evaluated. The LZ-SAA test kit was validated and verified in previous studies by Hansen *et al*. [21] and Tamamoto *et al*. [22]. No other test modality is available at present.

Another limitation was that the influence of concurrent diseases limited the comparability of SAA concentrations. The high prevalence of comorbidities in the present study and previous studies emphasizes the difficulty of recruiting cats without comorbidities and the requirement of a functioning biomarker that can be used even in cats with comorbidities. Imaging procedures excluded radiography (X-ray) and computed tomography (CT). Sonography was sufficient for the diagnosis of renal azotemia. X-ray and CT would not have provided any further relevant information and would have increased radiation exposure to the staff as the administration of anesthesia is considered too risky in patients with ongoing kidney damage. Finally, the diagnoses of AKI, CKD, and AoC were not confirmed through histopathological examination of the kidney tissue, which may have led to the misclassification of some cats. However, renal biopsy is an invasive procedure that cannot be performed if it does not have a sufficient therapeutic impact. Therefore, the diagnoses of AKI, CKD, and AoC were verified by board-certified internal medicine or emergency and critical care clinicians.

## Conclusion

Although SAA concentration increased in 44% of the azotemic cats, it cannot be used to differentiate between AKI, CKD, and AoC or as a prognostic marker. The reference range of the test kit limited the comparability of SAA concentration between the groups. Serum amyloid A concentration correlated with neutrophil count, albumin concentration, and UP/C but not with creatinine levels. Further studies are necessary to evaluate SAA concentration in cats without the possible contribution of comorbidities.

## Authors’ Contributions

LD, RD, RoD, KH: Planned and designed the experiment. LD and RD: Recorded and analyzed the samples and wrote the paper. RoD and KH: Edited the manuscript. All authors have read, reviewed, and approved the final manuscript.
